# The Effect of Natural Adjuvants on Pathological Changes in Sensitized Guinea Pig Lungs

**DOI:** 10.5812/ircmj.14267

**Published:** 2014-02-07

**Authors:** Ali Neamati, Mohammad Hossein Boskabady, Abass Tabatabaei, Saleh Mohaghegh Hazrati

**Affiliations:** 1 Department of Biology, Islamic Azad University, Mashhad Branch, Mashhad, IR Iran; 2 Neurogenic Inflammation Research Center and Department of Physiology, Mashhad University of Medical Sciences, Mashhad, IR Iran; 3 Department of Pathology, Mashhad University of Medical Sciences, Mashhad, IR Iran; 4 School of Public Health, Tehran University of Medical Sciences, Tehran, IR Iran; 5 Dr. Mohaghegh’s Foundation Researches on Industrial Biotech, Tehran, IR Iran

**Keywords:** Asthma, Lung, Pathology

## Abstract

**Background:**

Anti-inflammatory effect of natural adjuvants has been reported. Lung inflammation is the most characterized pathological feature in asthma.

**Objectives:**

The effects of three natural adjuvants (PC, G2, and G2F registered as a patent in the Iranian Patent Office) on sensitized guinea pigs lungs were examined in the present study.

**Materials and Methods:**

Lung pathological changes were examined in control and five groups of randomly divided guinea pigs including: sensitized animals (S, receiving normal saline, 0.5 ml i.p.); sensitized animals treated with adjuvant PC; G2F (0.1 ml i.p. for both cases); G2 (0.4 ml i.p.); and PC + G2 (receiving both PC and G) adjuvants (twice a week for 4 weeks for all groups). Sensitization of animals was done by injection and inhalation of ovalbumin (OA).

**Results:**

All pathological changes in S group including the eosinophil infiltration (scoring 3.28 ± 0.28), lymphocyte infiltration (2.82 ± 0.26), local epithelial necrosis (2.71 ± 0.47) and mucosal plug (2.75 ± 0.37) were significantly higher than control group (0.64 ± 0.18, 1.36 ± 0.24, 0.36 ± 0.18 and 0.28 ± 0.18 for eosinophil infiltration, lymphocyte infiltration, epithelial necrosis and mucosal plug respectively, P < 0.001 for all cases). Treatment with all adjuvants improved all pathological changes significantly (P < 0.05 to P < 0.001).

**Conclusions:**

These results indicate preventive effects of all natural adjuvants (especially G2) on pathological changes of the lung in sensitized guinea pigs.

## 1. Background

Asthma is an inflammatory disorder of the airway (1). The association between airway inflammation, epithelial damage, and airway hyper responsiveness has been reported in sensitized animals (2) and asthmatic patients (3). Many inflammatory cells involved in asthma producing more reactive oxygen species (4); which contract airway smooth muscle preparations directly and also appear to stimulate histamine release from mast cells and mucus secretion from airway epithelial cells (5). Sensitized guinea pig to OA is a well-documented experimental model of asthma. Increased tracheal responsiveness to methacoline and OA, a well-known characteristic feature of asthmas, as well as increased total white blood cells and eosinophil count, was shown in this model (6). In addition, it was shown that sensitization with OA can lead to immunological changes including increase in IL-4, but decrease in INF-γ and INF-γ/IL-4 ratio (7). Increased pathological changes in the lung including eosinophil and lymphocyte infiltration, ephithelial necrosis and mucosal plug were demonstrated in the guinea pig model of asthma induced by OA (8, 9). It is suggested that consumption of spleen extracts supports the immune system (10, 11). Researchers have shown that calf spleen extract reversibly and non-toxically inhibits lymphocyte reversible proliferation in specific tissues (12) and stimulates polymorph nuclear leukocyte activities (13).

The promotion of innate immune responses and atopy suppress; which include TH1-type patterns in individuals with early-life environmental exposure to microorganisms and their products were documented (14). Stimulation of lymphocyte functional activity and cytokine mRNA expression in swine by mycobacterium cell wall extract was also shown (15). Exposure to Mycobacteria or mycobacterial proteins led to reduction in different atopic manifestations (16). Intratracheal administration of Mycobacterium vaccae (M vaccae) ameliorating effect on airway histopathology features of a murine asthma model was also shown (17). PC Adjuvant is a bacterial extract polypeptide and its components include polypeptides proteins polysaccharides and phospholipids. G2 Adjuvant has been prepared from buffalo spleen lipid and its components include different kinds of lipids, glucose, cholesterol, and triglyceride. The stimulatory effects of PC and G2 adjuvants on Th-1 cells have been shown (18). They also have therapeutic effects on different illnesses such as allergy and asthma (19). The G2F adjuvant is the extract of Sesame Seed (*Sesamum indicum* L.). Since the early days, Sesame Seed has been known as one of the oldest seeds used for extracting cooking oil. Earliest findings regarding sesame dates back to 3000 years BC in the history of the Middle East Ancient athletes; which used it for enhancing their power and physical fitness. The sesame seed pure oil and/or its components have been used as adjuvant for different aims e.g. increase in vaccine potency (20) and cellular immunity (21) and natural killer cells activity (22); however researchers did not found any increase in antibody production (23). Moreover, Sesame oil has antioxidant activity, inhibits lipid per oxidation and might be used as a drug delivery system (24).

## 2. Objectives

In a previous study, the effect of PC and G2 on tracheal responsiveness and cell count in lung lavage of sensitized guinea pigs was shown (25). In the present in vivo study, we examined the effects of the PC, G2, PC+G2 and G2F adjuvants on lung pathological changes of sensitized guinea pigs.

## 3. Materials and Methods

### 3.1. Animal Sensitization

Sensitization of animals to OA was performed using the method described previously (26, 27). Briefly, guinea pigs were sensitized to OA (Sigma Chemical Ltd, UK) by injecting 100 mg i.p. and 100 mg s.c. on day one and a further 10 mg i.p. on day 8. From day 14, sensitized animals were exposed to an aerosol of 4 % OA for 18±1 days, 4 min daily. The aerosol was administered in a closed chamber, dimensions 30×20×20 cm. Control animals were treated similarly, but saline was used instead of OA solution. In an experimental study, thirty adult Dunkin-Hartley guinea pigs (550 to 700 g) of both sexes were divided into six groups of 5 animals using simple randomization method. These groups include: 1) control group; 2) sensitized animals (S) with OA alone (an animal model of asthma) receiving normal saline, 0.5 ml i.p. twice a week for 4 weeks; 3) sensitized animals treated with adjuvant PC, 0.1 ml i.p. twice a week for 4 weeks (S + PC); 4) sensitized animals treated with adjuvant G2, 0.4 ml i.p. twice a week for 4 weeks (S + G2); 5) sensitized animals treated with adjuvants PC and G2 (S + PCG2), and 6) sensitized animals treated with adjuvants G2F, 0.1 ml i.p. twice a week for 4 weeks (S + G2F). The study was performed in the Department of Physiology, Mashhad University of Medical Sciences, Mashhad, Iran in 2010 with code 85097, approved by the ethical committee of the University. Animals were housed in individual cages with access to standard food and water ad libitum and were maintained at 22º ± 2ºC on a 12 hr light/dark cycle (light period 0700 and 1900 hrs). Experiments were performed in compliance with the rulings of the Institute of Laboratory Animals Resources, Commission on Life Sciences, National Research Council (28).

### 3.2. Preparation of the Adjuvants

PC Adjuvant is registered as a patent in the Iranian patent office as: Immune system Activator vaccine (Innovation Register No: 36681, 28th of October 2006). Briefly, PC were prepared from Mycobacterium tuberculosis (M tuberculosis) H37Rv cultured in Long's medium with some modifications. Microorganisms with surface pellicles were cultured for 10 days at 37°C, and then re-cultured for 6 weeks. Microorganisms were then harvested and the killed particles were filtered in steam cabinet with different filters including Berkefeld filters. Phenol (0.35 %) + Trichloroacetic acid (40 %) were added to filtered materials and centrifuged at 850 × g for 30 min. The product was washed three times with KH2PO4 and centrifuged at 4000 × g for 30 min. The precipitated materials were dissolved in 0.5 % Na2HPO4 and clarified by high speed centrifugation at 15000× g at 4°C for 4 h. Supernatant was filtered in Berkefeld filter and added 0.5 % phenol and diluted up to 2.5 µg / ml (18, 19).

G2 is registered as a patent in the Iranian Patent Office as: Immune System Activator Vaccine (Innovation Register No: 36679, 28th of October 2006). Briefly, spleen were crashed in small pieces and diluted in the alcohol for few days, then were centrifuged at 800 × g for 30 min and supernatant were dried to get 20 µg/ml of concentrate (18, 19). For G2F preparation, the crude Sesame seed oil have prepared from the Esfahan state of the middle of Iran. The oil filtered with 0.5 µm filter paper to remove particles. The oil was then centrifuged at 10000× g at room temperature. The supernatant were filtered with 0.22 um pure size filters under vacuum filtration. Three hundred µg/ml of G2 was added to resulted preparation and called G2F and divided in 5 ml glasses under the sterile condition (18, 19). All three adjuvants are liposome and water soluble materials.

### 3.3. Pathological Evaluation

The lung tissues were prepared for histological evaluations as follows:

1. Fixation of tissues with formalin 10 %
2. Drying of tissues using Autotecnicon apparatus (tissue passage apparatus) by passage of tissues through ethanol 70 to 100 % and xylol to clear the tissues
3. Preparing paraffin block of the tissues
4. Preparing tissue slices with 3 to 5 µm thickness and putting them on a lam
5. Staining using standard Haematoxylin-eosin method.

The pathological changes of the lungs were included eosinophyl and lymphocytes infiltration, epithelial necrosis, and mucus plug which were scored as follows using light microscope by a pathologist; which was unaware of groups’ allocation:

- No pathological changes = 0
- Patchy pathological changes = 1
- Local pathological changes = 2
- Scatter pathological changes = 3
- Sever pathological changes = 4

### 3.4. Statistical analysis

The pathological changes of the lung were quoted as mean ± SEM. The sample size of the present study was chosen based on several previous studies (6-9), (25-27). According to the Kolmogorov-Smirnov test, the data of the present study had normal distribution. The data of sensitized group were compared with control guinea pigs using Student's t-test. The data of four groups of animals treated with the adjuvants were also compared with sensitized guinea pigs using Student's t-test. However, the data among four groups of animals treated with the adjuvants were compared using ANOVA with Tukey Kramer post-hoc test. Significance was accepted at p < 0.05.

## 4. Results

**Figure 1.**
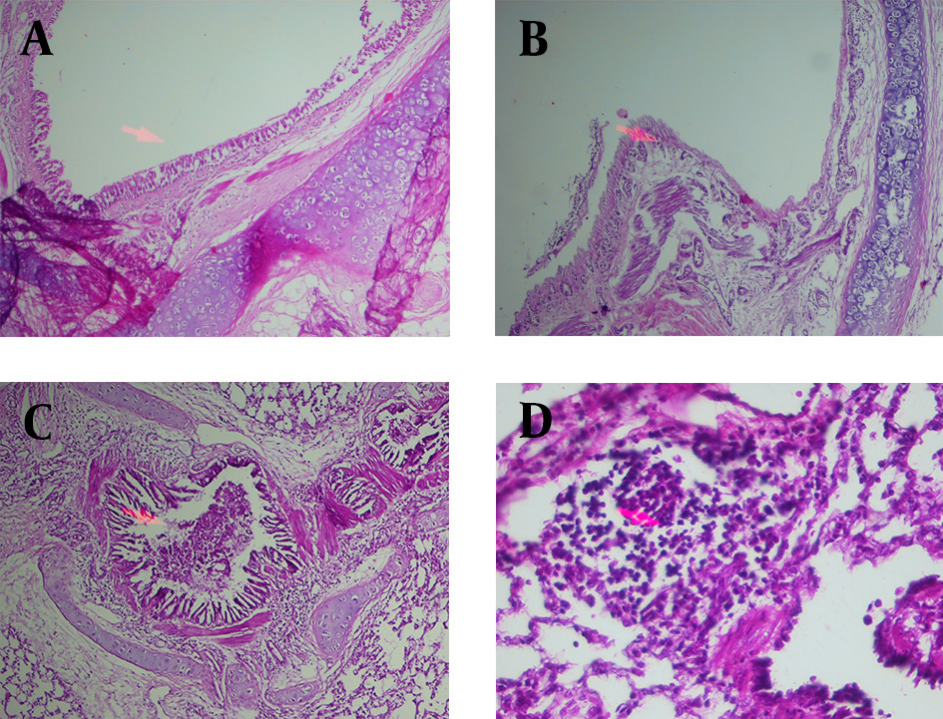
Photograph of a lung Specimen in Control Groups With Normal Epithelium (a), and Three Specimens in Sensitized Animals With Epithelial Shedding (b), Mucus Plug (c) and Inflammatory Cell Infiltration (d). The Magnification of Photograph a, b and c is 10 * 10 and that of d is 20 * 10

**Figure 2.**
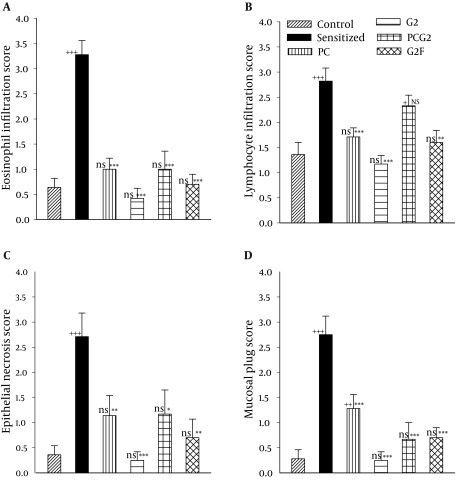
The Score of Eosinophil Infiltration (a), lymphocyte Infilteration (b), Epithelial Necrosis (c) Mucosal Plug (d) of lung in Control, Sensitized (S), S Treated With Adjuvant PC (S + PC), With Adjuvant G2 (S + G2), With Adjuvant PC + G2 (S + PCG2) and With Adjuvant G2F (S + G2F) Guinea Pigs (For each group, n = 5). Statistical differences between control and different groups: ns; non significant difference, +; P < 0.05, ++; P < 0.01, +++; P < 0.001. Statistical differences between S + PC, S + G2, S + PCG2 and S+ G2F vs sensitized group: NS; non significant difference,*: P < 0.05, **: P < 0.01, ***: P < 0.001. Only necrosis was significantly lower in S + G2 than S + PCG2 group (P < 0.01).

All pathological changes in S group including the eosinophil infiltration (scoring 3.28 ± 0.28), lymphocyte infiltration (2.82 ± 0.26), local epithelial necrosis (2.71 ± 0.47) and mucosal plug (2.75 ± 0.37) were significantly higher than the control group (0.64 ± 0.18, 1.36 ± 0.24, 0.36 ± 0.18 and 0.28 ± 0.18 for eosinophil infiltration, lymphocyte infiltration, epithelial necrosis and mucosal plug respectively, P < 0.001 for all cases, Figures 1 and 2).

Treatment with all adjuvants caused significant improvement in all pathological changes of sensitized animals except for lymphocyte infiltration in PCG2 group (P < 0.05 to P < 0.001, Figures 1 and 2). In addition only lymphocyte infiltration in PCG2 group and mucosal plug in PC group were significantly different with those in control group (P < 0.05 and P < 0.01 respectively, Figures 1 and 2).

Only lymphocyte infiltration in PCG2 group was significantly greater than that of G2 group (P < 0.01). Although all pathological changes score in G2 group were very close to control group and were lower than other treated groups; but the differences between treated groups were not statistically significant.

## 5. Discussion

Increased pathological changes in sensitized animals lung including infiltration of eosinophil and lymphocyte in the lung parenchyma, epithelial damage and mucosal plug was showed in the present study which was similar to the results of our previous study (29). In fact, the existence of eosinophil in lung parenchyma, epithelial damage and increased tracheal responsiveness was demonstrated in our previous study, using the same method of sensitization of guinea pigs (30). Pretreatment of S animals with adjuvants PC, G2, combination of the two adjuvants and adjuvant G2F prevented increased pathological changes of the lung in sensitized guinea pigs. In addition, preventive effect of G2 adjuvant on pathological changes of sensitized animals was greater than other adjuvant. Prevention of pathological changes by the adjuvants in sensitized guinea pigs indicated an important therapeutic effect of the adjuvants on asthma. The therapeutic effect of the adjuvants on sensitized animals is due to their anti-inflammatory property. Our previous study also showed preventive effect of PC, G2 adjuvants and their combination on tracheal responsiveness as the main characteristic feature of asthma. Furthermore, total and most differential white blood cell count specially eosnophil and IL-4 decreased, but enhanced IFN-γ in treated sensitized guinea pigs which the results of G2 adjuvant was superior (25). These findings also indicate anti-inflammatory effects of the studied adjuvants and support the results of the present study.

The possible mechanism of these adjuvants' effects is their antioxidant profile. The uptake of antioxidants may slow down the Th1-type immune response and thereby favors an overproduction of Th2-type cytokines (31). In fact, foods rich in antioxidants may cause a lack of triggers for the Th1-type immune response (32). In addition a relatively powerful preventive effect of vitamin C on increased tracheal responsiveness of sensitized guinea pigs with similar method of sensitization has been shown in our previous study (33). However, the effect of the adjuvants on regulation of Th1 and Th2 cells balance and their antioxidant effect should be investigated in further studies. Several studies examined the effect of different vaccine derived from bacteria products on asthma and showed beneficial treatment effect on this disease; which support the results of the present study. For example, researches reported that commensal gut microflora suppress allergic Th2 responses (34). Evidence shows that infection with M. bovis- Bacillus Calmette–Guérin (BCG) prevents airway eosinophilia, develops airway hyper responsiveness (AHR), and partially reduces the levels of allergen-specific IgE/IgG1 serum antibodies in the model of ovalbumin (OA)-induced allergic disease in mice, rats or guinea pigs (32, 35); which support the results of the present study. The BCG 16 weeks prior to OA airway challenge is able to inhibit bronchial eosinophilia, AHR and reduced IgE levels even in newborn Th2-susceptible hyper-IgE mice (36). Moreover, intranasal application of purified protein derivative from Mycobacterium tuberculosis is able to prevent the development of allergic rhinitis in mice (37). It was also reported that mycobacterial chaperonins and lipoglycans including the mycobacterial cell wall component ManLAM (mannose-capped lipoarabinomannan) have the ability to inhibit allergen-induced pulmonary eosinophilia in the murine model of allergic airway disease (26, 38); which also support the results of the present study because the PC2 adjuvants has liposacarid structure. Pathogen-associated molecular patterns (PAMPs) are widely conserved among different bacterial species; which play an important role in the activation of both innate and adaptive immune responses. Most - but not all - PAMPs bind to Toll-like receptors, expressed by a wide variety of immune cells, lead to cell activation (39). This mechanism may be also responsible for the observed effect of examined adjuvant in the present study. In a recent study, the leukocyte activation effect of fetal sheep liver extract containing trace amount of immunostimulatory substances including LPS in mice was shown (40). This study also supports the results of the present study because the studied adjuvants in our study also contained LPS substances. In another study, macrophage immunomodulatory activity of polysaccharides isolated from Juniperus scopolorum was observed (41); which can also support the data of our study.

The increase effect of G2F adjuvant (the extract of Sesame Seed) on cellular immunity (23) Natural Killer cell activity (22) and its antioxidant activity (24) has been shown. Therefore, the protective effect of G2F adjuvant is suggested to be due to its effect on immunity mechanisms or its antioxidant property. In fact, the effect of this adjuvant on inflammatory cells and cytokines my support its effect on immunity mechanisms. The antioxidant effect of the G2F adjuvant is supported by the results of our previous studies; which showed preventive effect of ascorbic acid (33) a and the extract of Nigella sativa (42, 43) as antioxidant agents. The preventive effect of G2 and G2F adjuvant on lung inflammation (total and differential WBC count) in sensitized guinea was also demonstrated (44).

However, the exact mechanism of action of different adjuvants on asthma and other inflammatory diseases and the contribution of different fractions (substances) of each adjuvant should be examined in further studies. The results indicated that G2 adjuvant has greater effects on pathological changes of sensitized animals. The differences in their preventive effect of different parameter of sensitized animals are perhaps due to variation in their chemical composition. The mechanism(s) of these differences should be also examined in further studies. In the present study, the preventive effect of different natural adjuvants on pathological changes of the lung of sensitized animals (an animal model of asthma) was shown; which is an important indicator of the studied adjuvants on lung inflammation effect in asthma. However, in further studies, the effect of the studied adjuvants on lung lavage and serum of sensitized animals and the effect of different adjuvant compositions on lung pathology and inflammatory mediators should be examined. Finally, the preventive effect of the adjuvants on asthmatic patients should be evaluated. 

Regarding to safety of these adjuvants, eight different doses of G2 adjuvant including 1, 2.5, 5, 10, 20, 40, 80, 160 μg were injected to mice (i.p.) for 18 weeks (n = 20 for each doses). There was only little death among studied animals (totally 18 animals in all groups). There was not any relationship between the number of dead animals and administered doses. For PC adjuvant, three concentrations including, 25, 30 and 35 were injected to mice (i.p) for 18 weeks (n = 20 for each doses). There was not any death among animals of three groups (unpublished data). In conclusion, the results of the present study indicated therapeutic effects of three different natural adjuvants and their combination on pathological changes of sensitized guinea pigs. The prevention effect of the adjuvants seems to be due to their anti inflammatory property.
